# Exogenous and endogenous microbiomes of wild-caught *Phormia regina* (Diptera: Calliphoridae) flies from a suburban farm by 16S rRNA gene sequencing

**DOI:** 10.1038/s41598-019-56733-z

**Published:** 2019-12-30

**Authors:** Jean M. Deguenon, Nicholas Travanty, Jiwei Zhu, Ann Carr, Steven Denning, Michael H. Reiskind, David W. Watson, R. Michael Roe, Loganathan Ponnusamy

**Affiliations:** 1 Department of Entomology and Plant Pathology, 3230 Ligon Street, Campus Box 7647, North Carolina State University, Raleigh, NC 27695-7647 USA; 2 Comparative Medicine Institute, North Carolina State University, Raleigh, NC 27695 USA

**Keywords:** Ecology, Microbiology

## Abstract

The black blow fly, *Phormia regina* (Meigen) (Diptera: Calliphoridae) is one of the most abundant carrion flies in North America. Calliphorids are important in agriculture and animal production, veterinary sciences, forensics and medical entomology. While the role of flies in the epidemiology of human and animal diseases is an active area of research, little is known about the microorganisms associated with these insects. We examined the diversity of wild-caught black blow fly endogenous (internal body) and exogenous (external body) microbial communities using 16S rRNA gene sequencing. Overall, 27 phyla, 171 families and 533 genera were detected, and diversity was significantly higher (*P* < 0.05) on external body surfaces. At the genus level, *Dysgonomonas*, *Ignatzschineria*, *Acinetobacter*, *Vagococcus*, *Myroides*, and *Wohlfahrtiimonas* were predominant. Cloning and sequencing of nearly full-length fragments of the 16S rRNA gene showed that some of the species identified are known to be pathogenic to humans, animals, and plants. *Myroides odoratimimus* and *Acinetobacter radioresistens* are well-known, multi-drug resistant bacteria. These results provide a snapshot of the microbial communities harbored by adult black blow flies and call for more comprehensive studies to better characterize the role these flies may play in the transmission of pathogenic microorganisms.

## Introduction

Blow flies (Diptera: Calliphoridae) are a group of medium to large flies with a worldwide distribution, usually harboring bright or metallic colors^1^. In calyptrate flies, calliphorids represent about 1,500 described species and account for 8% of species diversity^2,3^. They are saprophagous/necrophagous insects, breeding in decaying organic matter of plant and animal origins and are of medical^4–6^, veterinary/agricultural^7^, and forensic importance^8,9^. For example, adult calliphorids are known to be the first insects to colonize animal and human remains and are frequently used in the estimation of the minimum postmortem interval (minPMI) in medico-legal investigations^10^. Several species of blow flies, such as the primary screwworm, *Cochliomyia hominivorax* (Coquerel) and the Australian sheep blow fly, *Lucilia cuprina* (Wiedmann) (Diptera: Calliphoridae), are notorious myiasis (flystrike) agents in livestock causing hundreds of millions of dollars in economic losses^7,11^.

The presence of blow flies in microbe-rich substrates, such as manure, carrion and corpses, confer them a high probability of encountering/interacting with a large variety of microorganisms such as bacteria^12^, while their synanthropic nature (association with human environments) as well as strong flight abilities^13^, allow them to potentially disseminate those organisms over long distances into human habitat; they have the potential of infecting different substrates through contact by just landing on a surface but also by regurgitation of their gut content and defecation^14–16^. In addition, it has also been shown that blow flies can carry and mechanically transmit several bacterial pathogens, including *Burkholderia pseudomallei*^17^, *Providencia rettgeri*, *Morganella morganii*, *Klebsiella* spp. and *Staphylococcus aureus*^18^; *Escherichia coli*^19,20^; and *Helicobacter pylori*^21^.

The black blow fly, *Phormia regina* (Meigen) (Diptera: Calliphoridae), the focus of this paper, is a Holarctic fly and is distributed throughout the Northern Hemisphere^22^. In the USA, *P*. *regina* is typically a cold weather fly, as it is more abundant during the “chilled” summer months in the Northern part of the US while being more frequent in the cooler months of spring and fall in the South^23,24^. However, high population densities of *P*. *regina* have previously been recorded during the summer in Kansas^25^ and California^26^. *Phormia regina* has been of interest since it is one of the most abundant carrion flies in North America^27^ and is one of the primary insects used to estimate the minimum postmortem interval (minPMI) in forensic entomology^28^. *Phormia regina* is also listed by the U.S. Food and Drug Administration (FDA) as one of the 22 pests (the “Dirty 22”) that could potentially spread food-borne pathogens^29^ and is an important research model for the study of fly physiology^30–32^. Several studies have been conducted on *P*. *regina* morphology^33^, genomics^27^, biology and ecology^9,34–36^, including aspects of the interactions that exist between this species and bacteria^37^. However, studies that investigated the microbial communities in free-flying, wild-type adult *P*. *regina* using 16S rRNA gene sequencing are absent. Weatherbee *et al*.^37^ investigated the internal microbiome of *P*. *regina* collected from swine carcasses but focused on third-stadium larvae. Russell *et al*.^38^ investigated the presence of antibiotic-resistant bacteria in adult Calliphorids collected from piglet cadavers introduced into a forest environment but only identified the flies to the family level. Also, the study was based on culture-dependent techniques.

In the present study, we investigated the endogenous and exogenous bacterial microbiomes of wild-caught adult black blow flies collected in a suburban environment using high-throughput 16S rRNA gene amplicon sequencing. We hypothesized that (i) the microbial communities from these two regions of the fly body would be different, (ii) bacterial diversity on the outside will be higher compared to the gut and (iii) the flies collected will harbor known pathogenic bacteria.

## Material and Methods

### Fly collection, identification, and initial sample preparation

Adult blow flies (mixed sexes) were collected at the Lake Wheeler Road experiment station at NC State University (NCSU), Raleigh, North Carolina in November 2017. This field station contains several animal production systems including a feed mill laboratory, equine education center, poultry waste management facility, etc.) and plant-related units (an agroecology farm, soil & water research center, etc.). This experiment station is located close to the main NCSU campus and urban areas in the city. Blow flies are strong fliers and highly adapted to evasion making it difficult to collect them individually in flight or after they have landed, therefore, an effective alternative approach is trapping. Flies were collected at a dump site (35°43′50.54′′N, 78°42′13.52′′W) using a Captivator fly trap jug (Starbar Products, Schaumburg, IL, USA) that contained 900 ml of a Flies-Be-Gone fly attractant solution (Flies-Be-Gone, Toms River, NJ, USA) (Fig. S1). The jug was covered with window screening (Model FCS8678-M, Saint-Gobain ADFORS, Grand Island, NY, USA) to prevent fly access to the attractant solution, and the trap was placed beneath a partially screened pyramid. The 1.2 m × 1.2 m × 1.2 m pyramid was constructed with standard framing lumber, and the screen was attached to the upper half with staples. Attracted flies that entered the pyramid were trapped at the top in an inverted 1.9 L Rubbermaid food storage canister (Model #007420216, Walmart, Raleigh, NC, USA). To prevent contamination of the flies from the trapping container itself, this container was washed with detergent, rinsed with sterile water and then cleaned with 95% ethanol. To reduce possible fly-fly interactions and fly-trap-fly interactions from affecting the fly microbiome, flies were collected only one hour after placement of the fly trap and transferred to the laboratory immediately. Flies were placed individually in 1.5 ml sterile microcentrifuge tubes (USA Scientific, Ocala, FL, USA), and the surface microbiota was collected by rinsing each fly 5 times, each time in 200 µl of sterile phosphate-buffered saline (PBS, Cat #00-3000, Invitrogen, MD, USA). All washes per fly were pooled into a 2 mL sterile microcentrifuge tube. The pooled surface washes (per fly) as well as the adult flies were then stored at −40 °C until further use. The flies were identified based on morphological characteristics^39^.

### DNA extraction and quantification

Prior to endogenous microbial DNA extraction, each adult fly was surface sterilized using 70% ethanol (30 s) followed by 1% bleach in water (30 s) and then washed 5 times with sterile water. The final washes were pooled together for subsequent verification of sterility. DNA extraction from internal (12 endogenous samples) and external body (12 exogenous samples) extracts was modified from Ponnusamy *et al*.^40^. Briefly, the pooled exogenous PBS wash (1 mL) was lyophilized and reconstituted with 200 µL of TNE buffer. For endogenous sampling of the microbiome, each fly was transferred to a sterilized screw cap 2 mL microcentrifuge tube (Catalog # 02-681-344, Fisher Scientific, Pittsburgh, PA, USA) containing 10 sterilized 3 mm solid glass beads (Catalog #11-312 A, Fisher Scientific, Pittsburgh, PA, USA) each and was homogenized in 200 µL TNE buffer using the FastPrep FP120 system (Thermo Electron Corporation, Waltham, MA, USA) for 30 s. For lysis, we added 160 µL of lysis buffer 1 (TNE buffer [100 mM Tris, 0.2 M NaCl, 10 mM EDTA, pH 7.4] containing 20 µL of proteinase K and 20 µl lysozyme). The samples were then incubated at 37 °C for 1 h. After which 200 µl of lysis buffer 2 (1% cetyltrimethylammonium bromide [CTAB], 1.5 M NaCl, 0.5 M Tris-HCl [pH 8.0], 0.1 M EDTA [pH 8.0]) was added, and the samples were further incubated at 56 °C for 1 h. The use of the whole body to sample the endogenous microbiome allowed us to account for bacteria present internally but localized to organs other than the gut. DNA was isolated through phenol-chloroform extraction and ethanol precipitation, and the resulting DNA was resuspended in 100 µl of molecular grade water. The DNA samples were further purified using the Wizard DNA cleanup system (Promega, Madison, WI, USA). The DNA quality and quantity were assessed using a NanoDrop 1000 Spectrophotometer (Thermo Fisher Scientific, Waltham, MA, USA). The total genomic DNA was normalized to 50-100 ng/µl and stored at −40 °C until PCR amplification.

### 16S rRNA gene amplification and NGS sequencing

To characterize the bacterial communities present in the samples (see S1 File), the hypervariable V4 region of the 16S rRNA gene was targeted using the 515 F (GTGCCAGCMGCCGCGGTAA) and 806 R (GGGACTACHVGGGTWTCTAAT) primers^41^. The 16S rRNA sequencing libraries were constructed according to the Illumina’s 16S rRNA metagenomics sequencing library preparation protocol (Illumina, San Diego, CA, USA). During library preparation, the amplicon products were cleaned following each PCR round using magnetic beads from the AxyPrep Mag PCR Clean-up kit (AXYGEN, Big Flats, NY, USA), and the size of the amplicons was verified each time on a 1.5% agarose gel. One endogenous sample failed to generate PCR amplicons and was excluded. Illumina sequencing libraries were constructed for all 23 remaining samples (11 endogenous and 12 exogenous), and amplicon DNA concentration was measured with Quant-iT PicoGreen (Molecular Probes, Inc. Eugene, OR, USA). Final libraries were pooled in equimolar amounts. Illumina sequencing (250-bp paired-ends) was performed at the Microbiome Core Facility, School of Medicine, University of North Carolina, Chapel Hill, NC, USA.

### Bioinformatics and statistical analyses

The sequencing data were processed using the Quantitative Insights Into Microbial Ecology 2 (QIIME2) program^42^. Illumina paired-end sequence reads were first demultiplexed to assign the sequences to their sample of origin using the QIIME2 demux emp-paired command and then quality filtered using the DADA2^43^ algorithm as a QIIME2 plugin. Through the DADA2 pipeline, the paired-end reads were joined together, denoised, and chimeras, as well as residual PhiX reads, were removed. This results in the identification of all unique bacterial sequences called amplicon sequence variants (ASVs) which are technically equivalent to 100% OTUs but without errors^44,45^. Primers sequences were trimmed off the reads, but the sequences were not truncated to increase the chances of joining the paired-end reads efficiently. The 16S rRNA gene sequence variants (representative sequences) were assigned taxonomic classification using a pretrained Naïve Bayes classifier which was trained on the Greengenes database (gg_13_8, 99% OTUs, as recommended in QIIME2)^46^. Taxonomy was assigned using the Qiime feature-classifier classify-sklearn command. To calculate the statistical difference between the exogenous and endogenous groups, relative abundances of taxa with abundance higher than 1% (across all samples) were arcsine square root transformed, and a Welch two-sample *t*-test^47^ was run using abundances from each sample as replicates. Relative abundance data were imported from QIIME2 into the R statistical software^48^ for running the *t*-tests.

Prior to computing diversity metrics, the representative sequences obtained after DADA2 were aligned and masked to remove gaps, and a mid-point rooted phylogenetic tree was constructed using the QIIME2 FastTree 2 plugin^49^. To avoid biases due to sample-based variations in library sizes and to retain all of our samples in the subsequent analyses, each sample was rarefied to a depth of 18,000 sequences per sample. Alpha diversity was estimated by the Shannon index (integrates both richness and evenness)^50^, the number of observed OTUs (ASVs), and Faith’s phylogenetic index^51^. The statistical significance of alpha diversity between groups (exogenous and endogenous samples) was inferred using pairwise Kruskal-Wallis H-tests. Beta diversity was calculated using the weighted UniFrac^52^ metric, and a PERMANOVA test was run to determine if there was any statistical difference between the two treatment groups. The principal coordinate analysis (PCoA) result was visualized using EMPEROR^53^.

### 16S rRNA gene cloning and sequencing

The short-read length of sequences produced by the Illumina MiSeq platform limited taxonomic resolution during taxonomy assignment of the 16S rRNA gene sequences. To improve classification, especially for the most abundant taxa (relative abundance ≥10% across all samples), a nearly full-length fragment of the 16S rRNA gene was amplified using the universal bacterial primers, 27 f and 1492r^54^ following a protocol previously described in Ponnusamy *et al*.^55^. DNA from four fly samples that contained the highest abundances of the targeted taxa were used (Table 1). In order to construct the 16S clone libraries, the PCR products were verified for their sizes on a 1.5% agarose gel stained with ethidium bromide and purified using the AxyPrep Mag PCR Clean-up kit. Purified PCR products from each sample were cloned into the pGEM-T Vector (Promega) as per manufacturer’s instructions. Colonies were picked at random, and the presence of the insert was verified by amplifying the clones with the vector primers M13F (CCCAGTCACGACGTTGTAA AACG) and M13R (AGCGATAACAATTTCACACAGG). In total, 30 colonies with inserts were first Sanger sequenced using only the M13F primer, and the sequencing results were blasted against the BLASTn (Basic Local Alignment Search Tool) sequence database to identify homologous sequences and examine phylogenetic relationships. Then, four colonies (one per sample/targeted taxa) were further Sanger sequenced using two additional internal primers (520F, 968F or 518R, 984R) based on the direction in which the insert was cloned into the vector. Sanger sequencing for all samples was done at Eton Bioscience, Inc (Research Triangle Park, NC, USA).

**Table 1:** Overall relative abundances (%) across samples at the family level.

Sample IDs	Porphyromonadaceae	Xanthomonadaceae	Moraxellaceae	Enterococcaceae	Enterobacteriaceae	Pseudomonadaceae	Flavobacteriaceae
Fi2	73.56	18.04	0	0.48	0.305	0.02	0
Fi3	47.51	27.5	0	1.6	0.101	0.03	0
Fi4	51.41	26.69	0	1.62	0.038	0.04	0
Fi5	6.18	12.19	0	5.27	0.25	0.046	0.05
Fi6	65.01	19.89	0	0.6	0.379	0.04	0
Fi7	89.87	5.8	0	1.17	0	0.02	0
Fi10	46.34	27.9	0	1.78	0.061	0.03	0
Fi11	72.36	17.38	0	0.96	0.151	0	0
Fi12	92.26	4.87	0	0.58	0	0	0
Fi13	7.18	13.17	0	5.1	0.34	0.07	0.11
Fi14	91.73	5.04	0	0.77	0.032	0.012	0
Fo1	34.33	23.85	21.18	3.53	2.15	1.3	2.11
Fo2	6.99	45.27	12.04	6.96	8.85	4.2	1.15
Fo3	7.69	43.36	11.86	9.44	7.6	4.2	1.12
Fo5	32.05	24.72	20.37	3.58	2.46	1.6	2.53
Fo6	17.59	25.99	20.92	6.85	1.54	1.4	4.7
Fo7	32.68	23.65	21.44	3.19	2.26	1.4	2.51
Fo10	7.36	43.53	11.81	8.41	8.1	4.1	1.1
Fo11	17.14	25.92	20.91	6.5	1.7	1.6	4.9
Fo12	12.8	18.47	36.04	6.55	2.86	3	3.11
Fo13	14.46	19.7	32.72	6.26	2.86	3.65	3.05
Fo14	12.04	17.86	34.95	7.92	2.78	4.9	2.65
Fo15	8.49	16.83	12.69	9.14	13.34	10.6	4.12
Average all	36.83	22.07	11.17	4.27	2.53	1.83	1.44

Sample IDs: Fi2 to Fi14, internal body (endogenous) sample; Fo1 to Fo15 external body (exogenous) sample.

### Phylogenetic analyses of 16S rRNA gene clones

For each of the four colonies, each targeting a different taxon, the DNA sequences obtained using the M13F and two internal 16S rRNA gene primers were imported into the Molecular Evolutionary Genetics Analysis (MEGA version 7.0) software^56^. The sequences were aligned using the ClustalW program. Due to sequencing errors and low-quality reads towards the ends of the reads when using Sanger sequencing, a less error-bearing 16S rRNA gene DNA sequence was reconstructed by joining three DNA contigs (500-600 bp each) together using a minimum 100 bp overlap between the sequences. First, 16S rRNA gene sequences were screened for vector contamination using the NCBI VecScreen program and sequences that were identified to be of vector origin were removed. The final version of each 16S rRNA gene clone sequence was then blasted against the NCBI GenBank database using BLASTn. The 16S rRNA gene sequences were blasted against the NCBI 16S ribosomal RNA database on February 6, 2019. Clones with DNA sequences sharing 99-100% query coverage and at least 99% identity with GenBank sequences were assigned to that specific phylotype. Multiple alignments were performed using the ClustalW algorithm. The phylogenetic relationship between these clones and a selection of other closely related species was reconstructed using the kimura two-parameter model^57^ and neighbour-joining algorithms as implemented in the MEGA7 software package.

## Results

### Data summary

DNA extraction from pooled washes (from surface sterilization) yielded no bands on agarose gel after PCR amplification and thus was not sequenced. A total of 23 16S rRNA gene libraries from exogenous and endogenous *P*. *regina* extracts for each fly separately were subjected to Illumina paired-end sequencing. A total of 10,622,697 16S rRNA gene sequences were obtained. After quality filtering using DADA2, 7,684,848 reads remained with an average of 334,124 reads (minimum of 18,504 and maximum of 517,162). The remaining sequences were clustered into 2,452 sequence variants and assigned to 27 phyla, 171 families and 533 genera (with some further classified into species).

### Alpha and beta diversity measures

**Figure 1:**
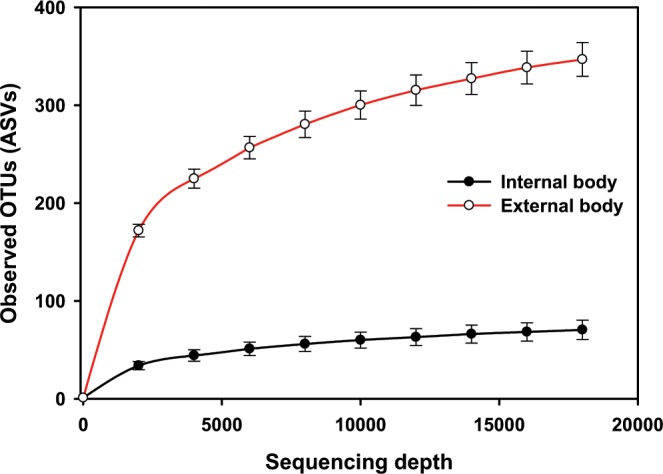
Rarefaction curves of the mean number of observed OTUs (sequence variants) in internal (endogenous) versus external body (exogenous) samples.

**Figure 2:**
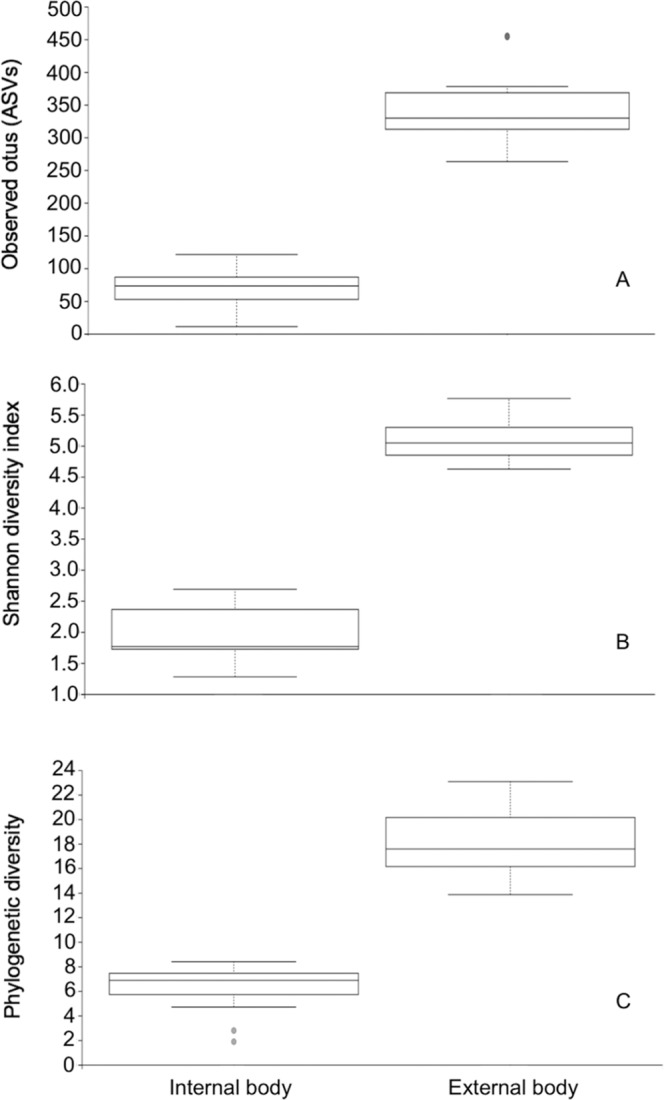
Alpha diversity measures of the internal (endogenous) and external body (exogenous) microbiomes of adult black blow flies. (**A)** Observed OTUs, (**B)** Shannon diversity and (**C)** Faith’s phylogenetic diversity.

The asymptotic shape of the rarefaction curves of the observed OTUs (ASVs) suggests that our sequencing depth of 18,000 was sufficient to capture the majority of taxa present in the samples (Fig. 1). Comparisons between outside and inside samples of the black blow flies revealed significant differences in alpha diversities. In fact, the number of observed ASVs was significantly higher from the outside samples compared to inside samples (Kruskal-Wallis, *P* < 0.001) (Fig. 2A). Similarly, Shannon diversity (Kruskal-Wallis, *P* < 0.001) (Fig. 2B) as well as phylogenetic diversity (Kruskal-Wallis, *P* < 0.001) (Fig. 2C) were significantly higher outside.

**Figure 3:**
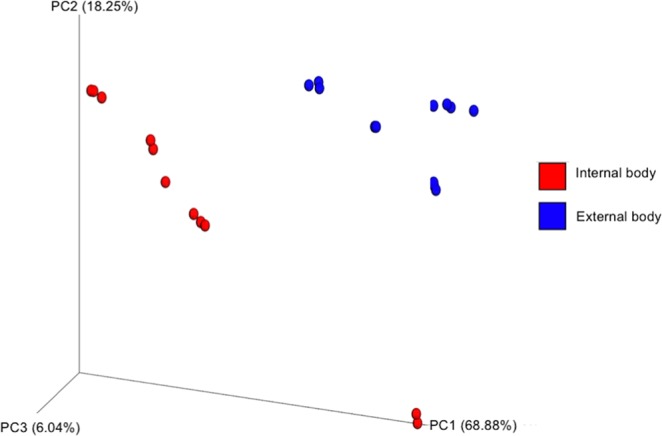
Principal coordinate analysis of bacterial composition between internal (endogenous) and external body (exogenous) samples of adult black blow flies. Analysis was based on the weighted Unifrac metric.

Bacterial community difference between the two groups (β-diversity of exogenous vs. endogenous communities) was tested using the weighted Unifrac metric. This analysis revealed that the majority of the variation in bacterial diversity across the samples could be attributed to where the samples originated from (Fig. 3). Principal coordinates analysis showed distinct clustering of exogenous and endogenous samples, suggesting differences in the microbial communities of these groups (Fig. 3). This was further confirmed by a permutation multivariate analysis of variance (PERMANOVA) which found a significant difference between these groups at the β-diversity level (PERMANOVA, *P* = 0.001).

### Bacterial community composition

**Figure 4:**
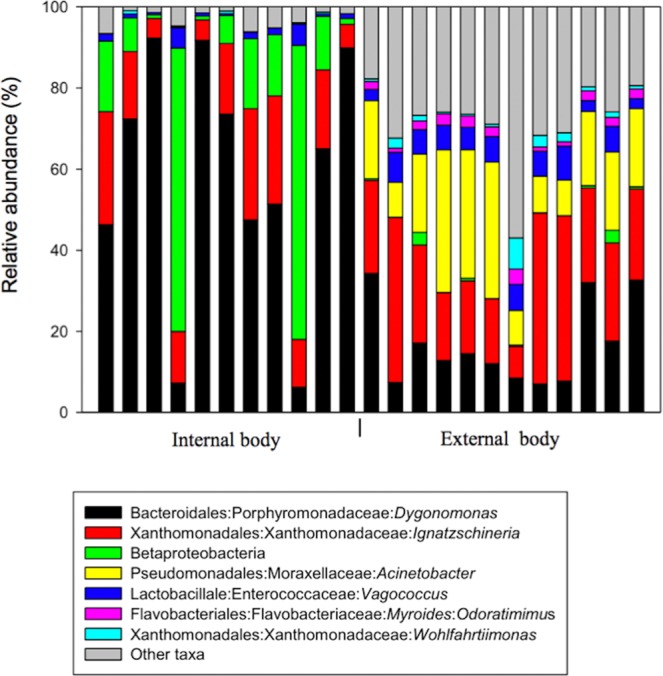
Relative abundance of major bacteria in internal (11 samples) and external body (12 samples) DNA samples from *P*. *regina* adults at the highest taxonomic resolution. Bars represent proportions of each taxa. “Other taxa” refers to all the taxa with relative abundance below 1% over the total number of reads. Ten per cent of the reads (10%) of the reads across all samples were classified as Betaproteobacteria. In the legend from left to right: Order:Family:Genus:Species.

Overall, the most sequence-abundant bacterial taxa at the phylum level were Proteobacteria (50.6%), Bacteroidetes (38.9%), Firmicutes (8.5%) and Actinobacteria (1.1%) (Fig. S2; see S2 File). Seven families were found to be the most sequence-rich and include Porphyromonadaceae (36.8%), Xanthomonadaceae (22.1%), Moraxellaceae (11.2%), Enterococcaceae (4.3%), Enterobacteriaceae (2.5%), Flavobacteriaceae (1.4%) and Pseudomonaceae (1.8%) (Table 1). At the highest taxonomic resolution (level 7, species level), most taxa were classified up to the genus level. In general, and across all samples, 6 genera were identified to be the most abundant: *Dysgonomonas* (36.8%), *Ignatzschineria* (20.6%), *Acinetobacter* (10.0%), *Vagococcus* (3.8%), *Myroides* (1.1%, further classified into species see Fig. 4) and Wohlfahrtiimonas (1.1%) (Fig. 4; see S2 File). These taxa were all differentially abundant between the two sites on the fly (Welch’s t-test, *P* < 0.05). In the exogenous samples, *Dysgonomonas* (16.9%), *Ignatzschineria* (24.9%), *Acinetobacter* (19.2%), *Vagococcus* (5.6%), *Myroides* (2.2%) and *Wohlfahrtiimonas* (1.8%) were the most abundant. The endogenous samples harbored *Dysgonomonas* (58.5%), *Ignatzschineria* (15.9%) and *Vagococcus* (1.8%), as the most abundant genera. *Myroides* (0.01%) and *Acinetobacter* (0.01%) were almost absent from the inside samples while *Wohlfahrtiimonas* had a very marginal relative abundance (0.3%) (Fig. 4). A bacterial taxon with 10% of the reads was classified only to the class level as a Betaproteobacteria (Fig. 4).

We also compared the bacterial diversity between individuals for the 7 predominant taxa found on the outside of the flies, the relative abundance of *Dysgonomonas* ranged from 7 to 34%; for *Ignatzschineria* ranged from 8 to 42%; for Betaproteobacteria ranged from 0.02 to 0.3%; for *Acinetobacter*, from 8 to 35%; for *Vagococcus*, from 2 to 8%; for *Myroides odoratimimus*, from 1 to 4%; for *Wohlfahrtiimonas*, from 0.4 to 8%. Furthermore, we detected several low relative abundance (<1%) bacteria (i.e. *Sphingobacterium multivorum*, *Faecalibacterium prausnitzii*, *Psychrobacter pulmonis*) found on some flies not found on others (see S2 File).

### 16S rRNA gene cloning and phylogeny results

All 30 16S rRNA cloned colonies that were Sanger sequenced using the M13 primers matched to their respective targeted taxa when the clone sequences were blasted against the NCBI database (http://blast.ncbi.nlm.nih.gov/). The nearly full-length reconstructed 16S rRNA gene clones had an average length of 1496 bp. The 16S rRNA gene clone sequence “Clone A1” matched to the species *Ignatzschineria ureiclastica* strain FFA (NR_116121.1) and *Ignatzschineria larvae* DSM 13226 (NR_025381.1) with 99% similarity. BLASTn searches with “Clone B9” affiliation to the species *Acinetobacter radioresistens* with 99% similarity to both the strains NBRC 102413 (NR_114070.1) and FO-1 (NR_026210.1). The first three highest BLAST matches for “Clone C8” were 16S rRNA gene sequences from *Dysgonomonas capnocytophagoides* strain JCM 16697 (NR_113133.1) (93%), *Dysgonomonas capnocytophagoides* strain CDC F9047 (NR_044778.1) (93%) and *Dysgonomonas alginatilytica* strain HUA-2 (NR_137388.1) (92%). The first four top hits after BLAST searches for “Clone D17” were: *Paraburkholderia rhizoxinica* strain HKI 454 (NR_102769.1) (94%), *Paraburkholderia rhizoxinica* strain HKI 454 (NR_042393.1) (94%), *Burkholderia rinojensis* strain A396 (NR_118637.1) (94%) and *Paraburkholderia endofungorum* strain HKI 454 (NR_042584.1) (94%).

**Figure 5:**
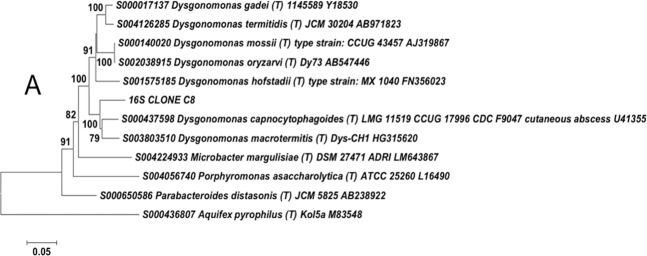
Neighbour-joining tree based on 16S rRNA gene sequences showing the relationship between cloned sequences from *Phormia regina* adults and sequences of other closely related bacterial species. Clone “16S CLONE C8” originated from sample (Fi12) that contained 92% of sequences assigned to the genus *Dysgonomonas*. *Aquifex pyrophilus* was used as an outgroup. The sequences were aligned using the ClustalW algorithm. Bootstrap values based on 500 replications, are given at the branching nodes. Bar represents 0.05 substitutions per nucleotide position.

The sequences used for constructing the trees were type species retrieved from the Ribosomal Database Project hierarchy browser (RDP; http://rdp.cme.msu.edu/)^58^. The 16S “CLONE A1” was placed within the genus *Ignatzschineria* (bootstrap value = 100) and more similar to *Ignatzschineria larvae* (Fig. S3). Clone B9 was more closely related to *Acinetobacter radioresistens* (bootstrap value = 100) within the *Acinetobacter* genus (Fig. S4). The placement of Clone C8 within the genus *Dysgonomonas* was well supported (bootstrap value = 100) and was more closely related to both *Dysgonomonas capnocytophagoides* and *Dysgonomonas macrotermitis* (Fig. 5). The phylogenetic tree revealed that the cloned sequence “Clone D17” was placed in a completely different clade from the genus *Burkholderia sensus lato* (Fig. S5).

## Discussion

In this study, we investigated, for the first time, the bacterial microbiome associated with the internal body (endogenous) and external body surfaces (exogenous) of *Phormia regina* adults collected from the wild in a suburban area but associated with a multi-use animal and Ag research production system in the NCSU campus. The study revealed the presence of several bacterial taxa that were, most of the time differentially abundant between the two environments. The results also showed that these flies harbored an appreciable number of potentially pathogenic as well as several potentially multi-drug resistant bacteria.

Our findings revealed the number of observed ASVs (OTUs), Shannon diversity, and Faith’s phylogenetic diversity were all significantly higher in the outside samples representing DNA extracted from the surface of each fly. This demonstrates that diversity was higher on the external surface of the fly’s body compared to the gut and therefore the flies harbored more different bacterial species on the outside of their bodies. This result was not unexpected and has been shown previously in flies. For example, using culture-dependent techniques, Adeyemi *et al*.^59^ demonstrated that both the number and variety of bacteria isolated from external surfaces of filth flies were significantly higher compared to the gut. One possible reason for higher diversity on the fly surface is the availability of high microbe-laden feeding/breeding sites; black blow flies are known for example to frequently visit human and/or animal dung^60^. In addition, blow flies, house flies, and flesh flies have adhesive pads between the tarsal claws of their legs called pulvilli which are covered in fine hairs or “tenant setae”. The setae have been shown to secrete an adhesive substance to increase surface area for attachment to smooth surfaces but are also deemed to retain many microorganisms on the legs^21,61–63^. Microorganisms also attach to other body parts such as the wings, the mouthparts and abdomen^21,62^.

Although the transfer of bacteria between flies during the trapping period cannot be ruled out, there was no indication that the trapping method was an issue. Alpha diversity and bacterial community composition analyses showed that the exogenous microbiomes were not homogenous between the flies; variability was determined to have resulted from differences in bacterial composition and taxonomic abundances between samples. Additionally, minor taxa (abundances <1%) included several bacterial species that were detected on some flies while absent (relative abundance of 0%) on others, suggesting that the exogenous samples were independent. Further, the timing (November) and the methods by which trapping was conducted permitted the collection of a single species (*Phormia regina*). Trapping of a single species mitigated potential contamination of our target flies by bacteria associated with other flies.

As shown in the PCoA plot for the abundance-weighted UniFrac distance, the exogenous samples formed a cluster that is separated from the endogenous samples. This could be due to the differential abundance of some important bacterial taxa between the two environments but also to the presence/absence aspects of those taxa from one environment to another (discussed more later).

Overall, Proteobacteria, Bacteroidetes, Firmicutes and Actinobacteria were the predominant phyla in our samples. This result is in agreement with findings in houseflies^64^, fruit flies^65^, flesh flies^66^ and other blow flies^21,37,67^. It is worth noting that the overall average relative abundances of the last four families were essentially driven by outside samples as members from those families were barely detected in inside samples (abundances ranging between 0 and 0.15%). No Moraxellaceae was detected inside. A similar trend was also observed by Weatherbee *et al*.^37^ who investigated the internal microbial communities of third-instar specimens of *P*. *regina* as well as the external microbiome of the larval mass (larva aggregate composed of multiple fly species including *P*. *regina*) present on swine carcasses at different time points. At 132 and 156 h after carcass placement, where *P*. *regina* larvae were the most predominant species (over 85% of the larval mass), several taxa at the family level identified outside were not detected within the larvae. These results suggest that both the immature and adult stages of *P*. *regina* may selectively control the gut bacteria either through digestion or other unknown mechanisms. Several families (Bacteroidaceae and Bifidobacteriaceae) identified among the 10 most abundant families in the swine carcass study were marginally present in our samples while Porphyromonadaceae, the most abundant family taxon found internally in this study, was not present and/or abundant within *P*. *regina* larvae. These differences may be due to several factors including developmental stage-related differences (adults and larvae may be differentially associated with certain types of bacteria), the geographic locations of the studies (Indiana vs. North Carolina) as well as the feeding sources available to the flies and different bacteria or differences in bacteria abundance in the available feeding sources. All those factors have been shown to significantly impact the microbiomes of several insects^68^.

At the genus level, *Dysgonomonas*, *Ignatzschineria*, *Acinetobacter*, *Vagococcus*, *Myroides*, and *Wohlfahrtiimonas* were the most predominant taxa in our study. These genera, even though their relative abundance may vary, have been isolated from various insect species before. The majority of them, however, are frequently detected together (in 16S-based NGS studies) and often times in high abundances in insects associated with filth and most particularly in saprophagous and/or necrophagous insects/flies^64–67,69,70^. This result may be due to similar ecological niches in those insects and their overlapping range of feeding sources, an adaptation of these bacterial taxa to physicochemical conditions of the guts of these insects and to the role that these microorganisms may be playing in host nutrition and/or fitness.

*Dysgonomonas*, the genus with the highest abundance has been isolated from a burying beetle *Nicrophorus vespilloides* (Herbst) (Coleoptera: Silphidae)^69^, a bombardier beetle *Brachinus elongatus* (Chaudoir) (Coleoptera: Carabidae)^45^, a wood-feeding termite *Reticulitermes speratus* (Kolbe) (Isoptera: Rhinotermitidae)^71^, the common house fly *Musca domestica* (Diptera: Muscidae)^64^, the oriental latrine fly *Chrysomya megacephala* (Diptera: Calliphoridae)^67^, the Australian sheep blow fly, *Lucilia cuprina* (Wiedmann) and the common green bottle fly *Lucilia sericata* (Meigen) (Diptera: Calliphoridae). Similarly, results from analyses of this genus isolated from termites, dung beetles, black soldier flies and mosquitoes suggest that *Dysgonomonas* is implicated in the breakdown of carbohydrates namely cellulose and hemicellulose as well as the production of vitamin B12^72–76^. Whether *Dysgonomonas* plays similar roles in the black blow fly is yet to be confirmed. However, the high abundance of this genus in internal samples in this study points to that direction.

*Ignatzschineria*^77^ and *Wohlfahrtiimonas*^78^ were both originally described in the larvae of a parasitic fly, the spotted flesh fly *Wohlfahrtia maginifica* (Schiner) (Diptera: Sarcophagidae) a major myasis agent in animals^66^. Phylogenetically, they are two related genera but belong to two different lineages. These two genera have been isolated from other flesh flies^66,79^, house flies^64^, blow flies^67,70^ and carrion beetles^69^. Little is known about the role that members of these genera play in insects. However, they both exhibit strong chitinase activity, and Toth *et al*.^77^ have suggested a possible role of *Ignatzschineria* in metamorphosis. The role that they play in adult insects needs investigation. The genus *Vagococcus* was previously isolated from fruit flies^80^, house flies^64^, blow flies^21,70^, red imported fire ants, *Solenopsis invicta*^81^ and burying beetles^69^. They are deemed to produce antiviral metabolites in *Aedes albopictus*^82^, but nothing is known about their role in *Phormia regina*.

*Acinetobacter* was almost exclusively located externally (abundance inside was 0.01%). In fact, *Acinetobacter* spp. are widespread in natural environments and thus are frequently recovered from soil, polluted water, plants, animals and humans^83^. However, they have also been isolated from the guts of different insects^84,85^. For example, Coon *et al*.^86^, showed that the inoculation of *Acinetobacter* spp. restored larval development in axenic mosquito larvae while Minard *et al*.^87^ suggested they aided in the digestion of plant nectar consumed by *Aedes albopictus* mosquitoes. It was also suggested that they may help detoxify defensive plant compounds for tropical herbivorous beetles^88^. They also secrete antiparasitic compounds in the guts of several insects^89^. The presence of this genus on the outer body of the flies is probably of environmental origin; they may have been picked up mechanically from a contaminated source and may not have a function role in fly biology. It would be interesting to investigate if the bacteria could sustain the black blow fly gut environment and what possible role those bacteria could play.

*Myroides odoratimimus*, previously known as *Flavobacterium odoratum*^90^, was also exclusively observed in outside samples (abundance inside was 0.01%). Like some of the previous taxa described in this section, *M*. *odoratimimus* are ubiquitous in natural environments and are commonly found in soil, fresh and marine water, meat-processing plants and insects^21,90,91^. *Myroides* spp. are well known for their ability to secrete antimicrobial substances^90^, and thus it is possible that they help protect insects that harbor them against other pathogenic bacteria.

Based on our findings in *P*. *regina*, the absence of some taxa on the outside of the fly that are found internally and the fact that the most abundant taxa found inside are also present on the fly’s surface suggests that the microbial communities associated with black blow flies are acquired from the environment probably from feeding/breeding sites. Also, based on previous fly research, it is likely that the internal bacteria present may contribute to the host’s development. Most of the bacterial taxa identified in the *P*. *regina* microbiome contained known pathogens (discussed in more detail below).

Most reads (89%) obtained from Illumina sequencing were classified down to the genus level even for the most sequence-rich taxa (except for Betaproteobacteria and *Myroides odoratimimus*) thus limiting the depth of analysis of the results. A literature search revealed that some of those genera are well known to be pathogenic microorganisms. To improve taxonomic resolution and to verify the detection of potential pathogens in our samples, the first four identified taxa in terms of relative abundance (≥10%, *Dysgonomonas*, *Ignatzschineria*, *Betaproteobacteria and Acinetobacter*) were further targeted by developing nearly full length 16S rRNA gene clones and sequencing them using Sanger sequencing. The results showed some of the most abundant taxa in our samples may be new bacterial taxa and also confirmed the existence of potential human, animal and plant pathogens in our fly samples.

Our results suggest that some of our samples contained a new genus in Burkholderiaceae: Ten percent of the reads was classified as Betaproteobacteria, a class in the phylum Proteobacteria. BLASTn searches for “Clone D17” which was targeting that class for better identification revealed that the clone sequence was 94% similar to two strains of *Paraburkholderia rhizoxinica*, a strain of *Burkholderia rinojensis* and a strain of *Paraburkholderia endofungorum*. During phylogenetic analyses, Clone D17 was placed in a completely different clade from the genus *Burkholderia* *sensus lato* (s.l.). Therefore, the bacterium corresponding to that sequence constitutes a distinct lineage with no closely related allied 16S rRNA gene sequences and probably represents a novel genus in the family Burkholderiaceae, order Burkholderiales, class Betaproteobacteria in the phylum Proteobacteria. The genus *Burkholderia sensus lato* (s.l.) is the original genus described by Yabuuchi *et al*.^92^. Since then, the taxonomy of this genus has been evaluated several times with the addition of several new taxa, and today it is subdivided into 5 different groups or “sub-genera”^93,94^. The most interesting issue is that during the phylogenetic tree reconstruction, we made sure to include at least one member from each of the five groups (based on type species present in RDP), but Clone D17 was placed as a sister group to *Burkholderia* and *Pandoraea* with strong support (bootstrap value = 93). No assumptions can be made at the current stage about the pathogenicity of this potentially new genus.

Our results indicate that our samples contained a new *Dysgonomonas* species: The taxa previously classified as *Dysgonomonas* was targeted with Clone C8. The sequence from this clone had 93% similarity with two strains of *Dysgonomonas capnocytophagoides* and 92% similarity with a strain of *Dysgonomonas alginatilytica* based on BLASTn searches. Phylogenetically, it clustered with all the other *Dysgonomonas* (bootstrap value = 100) but was more closely related to both *Dysgonomonas capnocytophagoides* and *Dysgonomonas macrotermitis* with strong statistical support (bootstrap value = 100). These results suggest that this molecular isolate represents a potential new species within the genus *Dysgonomonas*, family Porphyromonadaceae, order Bacteroidales, class Bacteroidia in the phylum Bacteroidetes. *Dysgonomonas capnocytophagoides*, formerly known as CDC group DF-3 (Center for Disease Control group dysgonic fermenter-3) is an opportunistic bacterium associated with diarrhea and bacteremia^95,96^. *Dysgonomonas macrotermitis*, on the other hand, was only recently isolated from a fungus-growing termite^72,97^ and has not been associated with any disease so far. More work is needed to better characterize the bacterium represented by Clone C8 (this study).

New *Ignatzschineria* species: The sequence from “Clone A1” matched to the species *Ignatzschineria ureiclastica* and *Ignatzschineria larvae* with 99% similarity based on BLASTn searches conducted in February 2019. Clone A1 clustered together with all the species in the genus *Ignatzschineria* (bootstrap value = 100) in the phylogenetic analyses but was more closely related to *Ignatzschineria larvae* (bootstrap value = 75) which can cause bacteremia^98^. This finding is indicative of a potential new species in the genus *Ignatzschineria*, family Xanthomonadaceae, order Xanthomonadales, class Gammaproteobacteria within the phylum Proteobacteria.

*Acinetobacter radioresistens:* Both BLASTn searches and phylogenetic analyses revealed the sequence of Clone B9 belonging to the bacterium, *A*. *radioresistens*. It was originally isolated from cotton and soil^99^. Like several other members of the genus *Acinetobacter*, this species has emerged as an opportunistic human pathogen. It can cause septicemia^100^, nosocomial bloodstream infection and community-acquired infections in immune-compromised patients^101,102^ and has the potential to damage human epithelial kidney cells through the production of outer membrane vesicles (OMVs)^103^. *A*. *radioresistens* have also been shown recently to cause nosocomial bacterial infection outbreaks in research animals^104^. Another concerning fact about this species is its ability to withstand harsh environmental conditions as well as its strong and well-established resistance to several antimicrobials. In fact, *A*. *radioresistens* is highly resistant to desiccation and radiation^105^ and is known to be resistant to Carbapenem, a class of antibiotic normally highly effective against multidrug-resistant bacteria^102,103,106,107^.

*Myroides odoratimimus*: This bacterium is also an opportunistic pathogen causing a variety of diseases/infections of which some are life-threatening. *M*. *odoratimimus* can cause bacteremia, endocarditis, pericarditis, pneumonia and septic shock, urinary tract infections, calcaneal ulcer and several other important bacteria-related infections in humans^64,66,108–111^. This species can also cause lethal infections in fish^112^.

In addition to the taxa reported here as the most abundant (≥1%), it is important to note the detection in our samples of *Pseudomonas viridiflava* (relative abundance of 0.7%), a multi host plant pathogen. *P*. *viridiflava* causes foliar and stem necrosis as well as stem and root rot in a wide range of plants such as tomato, melon and eggplant^113–115^. Finally, in insects, *Wohlfahrtiimonas chitiniclastica*, the most frequently identified species of the genus *Wohlfahrtiimonas* is known to cause human infections^21,66^.

In summary, this study provides, for the first time, a snapshot of the bacterial communities harbored both internally and externally by free-flying, black blow flies. The results revealed that the flies harbored a higher diversity of bacterial species on their body surfaces probably owing to their ecology and behavior. Even though the majority of the most abundant bacterial taxa identified in this study have also been reported in other flies/insects, the presence of many potentially new taxa further confirms the necessity to conduct microbiome studies in insects in general but more importantly in synanthropic insects that breed in microbe-rich substrates such human and animal dung. Arguably, the most intriguing fact is the identification of known human, animal and plant pathogens such as *Myroides odoratimimus*, *Acinetobacter radioresistens* and *Pseudomonas viridiflava* on the fly body of which several are also resistant to several antibiotics.

## Supplementary information

Supporting Information.

Supporting Information.2

Supporting Information.3

## Data Availability

The next-generation sequencing data associated with this study have been deposited in GenBank under SRA accession: PRJNA554278. Cloned sequences have been deposited in GenBank under accession numbers: MN172461-MN172464.
