# Supplementary material for: Exogenous and endogenous microbiomes of wild-caught Phormia regina (Diptera: Calliphoridae) flies from a suburban farm by 16S rRNA gene sequencing
Source: Sci Rep. 2019 Dec 30;9:20365. doi: 10.1038/s41598-019-56733-z (PMC6937299; doi:10.1038/s41598-019-56733-z)
Supplement: Supplementary file 1 — Supporting Information. [file 41598_2019_56733_MOESM1_ESM.docx]

For submission in:

Scientific Reports

**Exogenous and endogenous microbiomes of wild-caught *Phormia regina* (Diptera: Calliphoridae) flies from a suburban farm by 16S rRNA gene sequencing**

Jean M. Deguenon^1^, Nicholas Travanty^1^, Jiwei Zhu^1^, Ann Carr^1^, Steven Denning^1^, Michael H. Reiskind^1^, David W. Watson^1^, R. Michael Roe^1,2^, and Loganathan Ponnusamy^1,2*^

^1^Department of Entomology and Plant Pathology, 3230 Ligon Street, Campus Box 7647, North Carolina State University, Raleigh, NC 27695-7647, USA

^2^Comparative Medicine Institute, North Carolina State University, Raleigh, NC, 27695, USA

^*^Corresponding author’s email: [lponnus@ncsu.edu](mailto:lponnus@ncsu.edu)

**Supplemental figure legends**

**Fig. S1.** Fly trap used to capture adult black blow flies in the field. **A)** Jug containing 900 ml of Flies-Be-Gone fly attractant solution, **B)** Screened jug for fly access inhibition, **C)** Canister used to hold trapped flies, **D)** 1.2 m x 1.2 m x 1.2 m screened pyramid to force direction of attracted flies into the canister.

**Fig. S2.** Mean relative abundance (across all samples) of major bacteria in internal (inside) and surface (outside) DNA samples from *P. regina* adults at the phylum level. Values represent proportions of each taxa. “Other phyla” refers to all phyla with relative abundance below 1% over the total number of reads.

**Fig. S3.** Neighbour-joining tree based on 16S rRNA gene sequences showing the relationship between cloned sequences from *Phormia regina* adults and sequences of other closely related bacterial species. Clone “16S CLONE A1” originated from sample (Fo2) that contained 42% of sequences from *Ignatzschineria* based on QIIME/GreenGenes classification of Illumina MiSeq data. *Sharpea azabuensis* was used as an outgroup. *Listeria fleischmannii* was used as an outgroup. The sequences were aligned using the ClustalW algorithm. Bootstrap values based on 500 replications, are given at the branching nodes. Bar represents 0.05 substitutions per nucleotide position.

**Fig. S4.** Neighbour-joining tree based on 16S rRNA gene sequences showing the relationship between cloned sequences from *Phormia regina* adults and sequences of other closely related bacterial species. Clone “16S CLONE B9” is from sample (Fo12) that harbored 35% of *Acinetobacter* sequences. *Aequorivita viscosa* was the outgroup bacteria. The sequences were aligned using the Clustal W algorithm. Bootstrap values based on 500 replications, are given at the branching nodes. Bar represents 0.05 substitutions per nucleotide position.

**Fig. S5.** Neighbour-joining tree based on 16S rRNA gene sequences showing the relationship between cloned sequences from *Phormia regina* adults and sequences of other closely related bacterial species. Clone “16S CLONE D17” originated from sample (Fi5) that contained 72% of sequences assigned to the Class Betaproteobacteria. *Listeria fleischmannii* was used as an outgroup. The sequences were aligned using the Clustal W algorithm. Bootstrap values based on 500 replications, are given at the branching nodes. Bar represents 0.05 substitutions per nucleotide position.


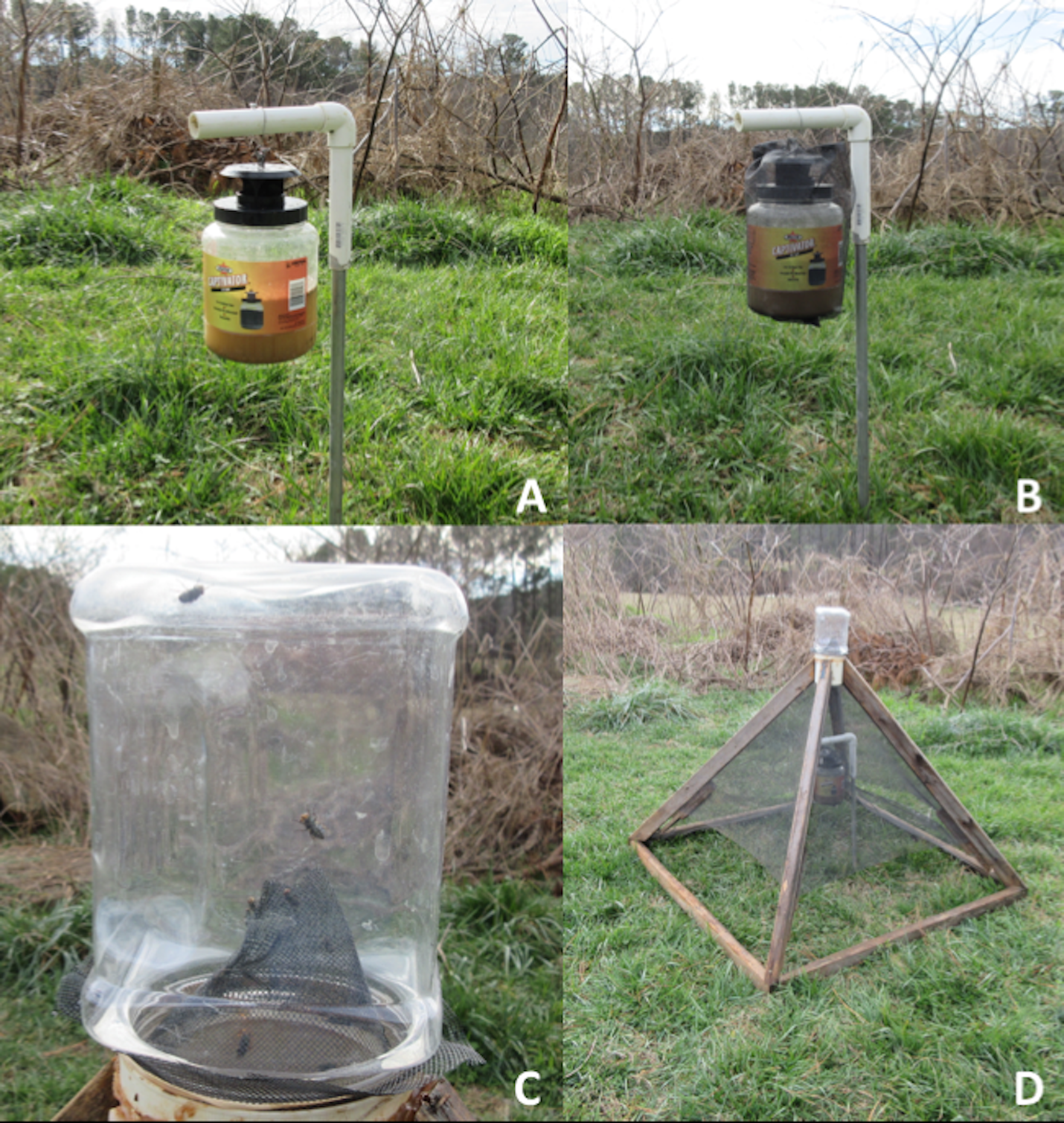


**Figure S1**


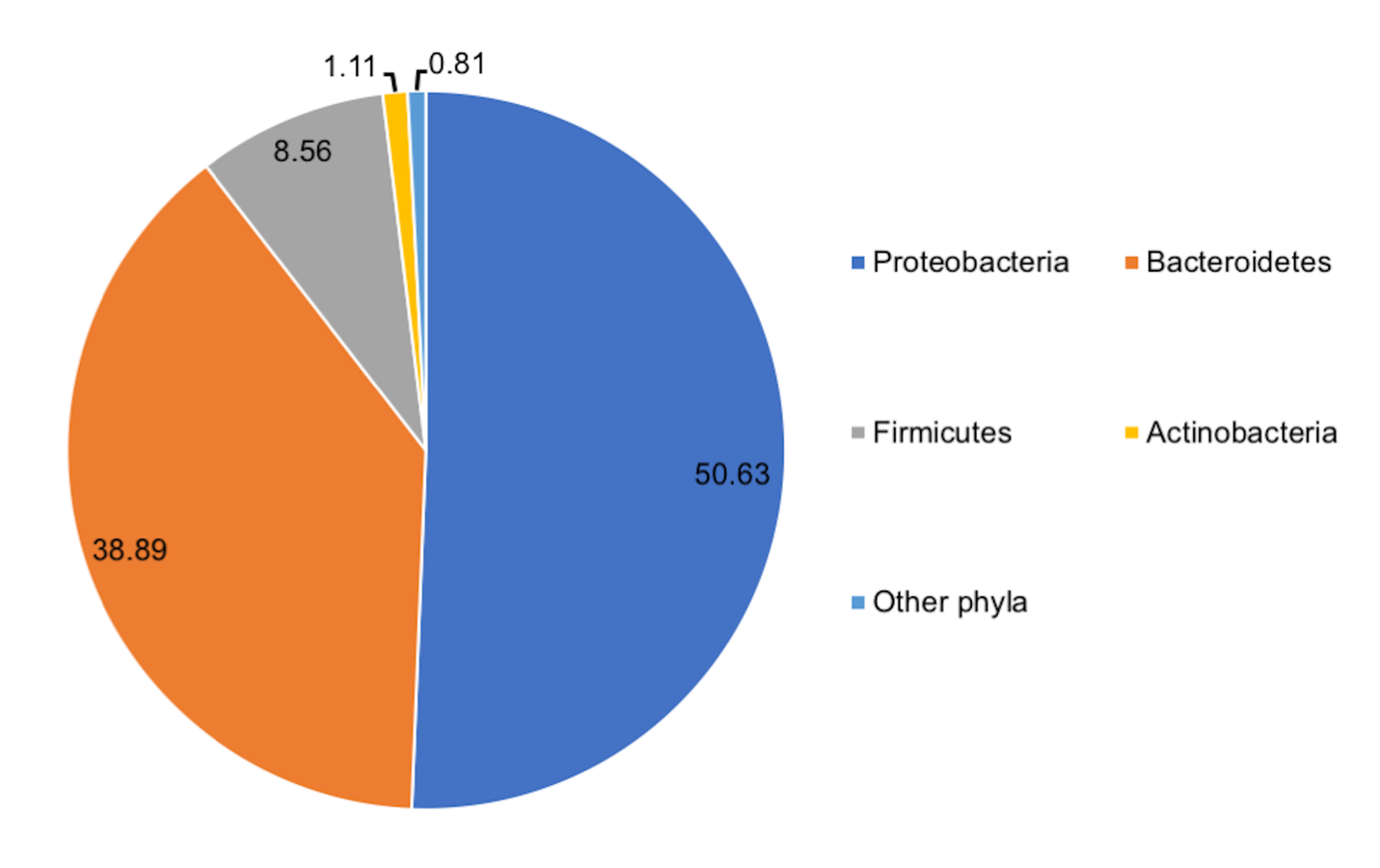


**Figure S2**

**
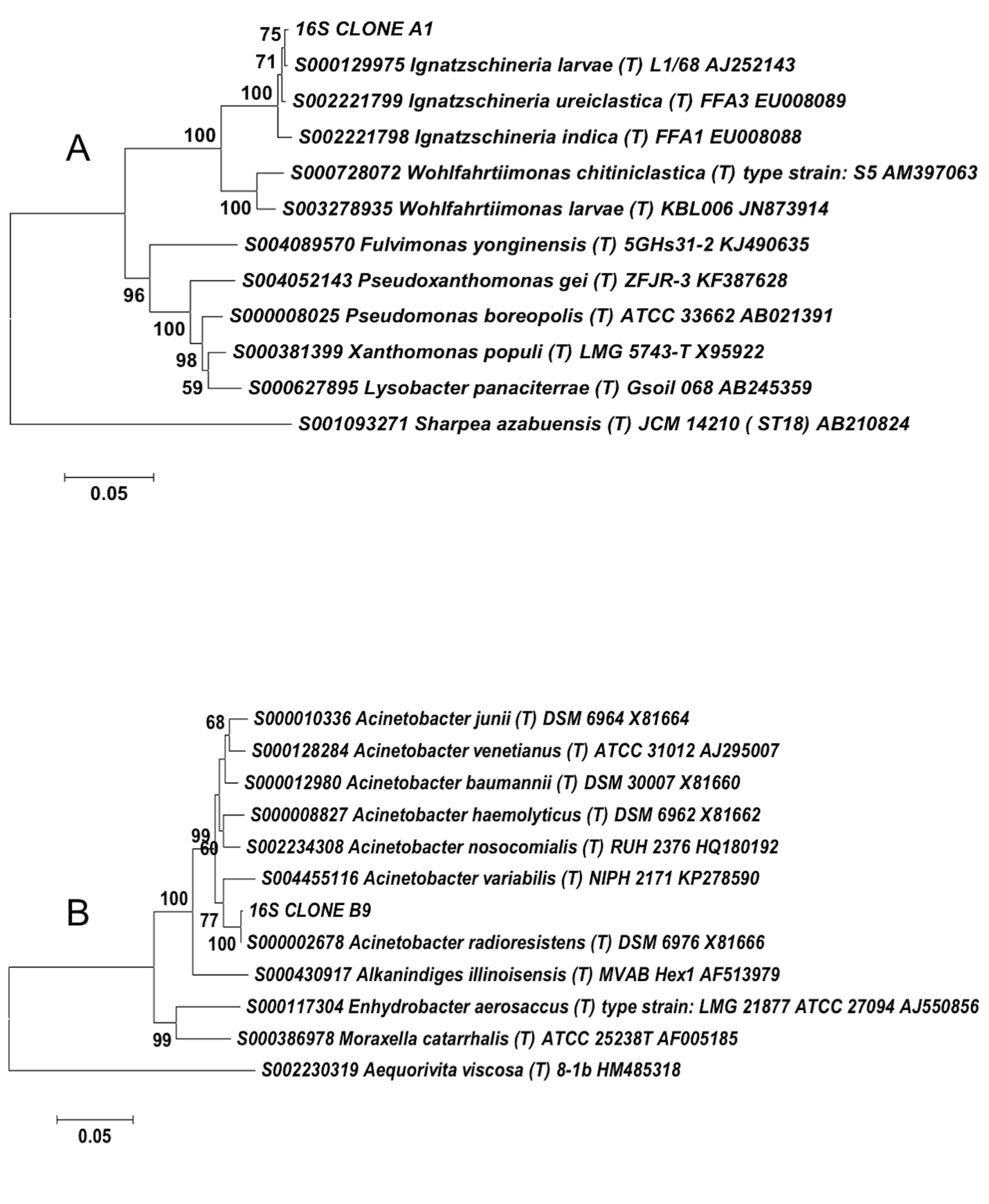
**

**Figure S3**

**
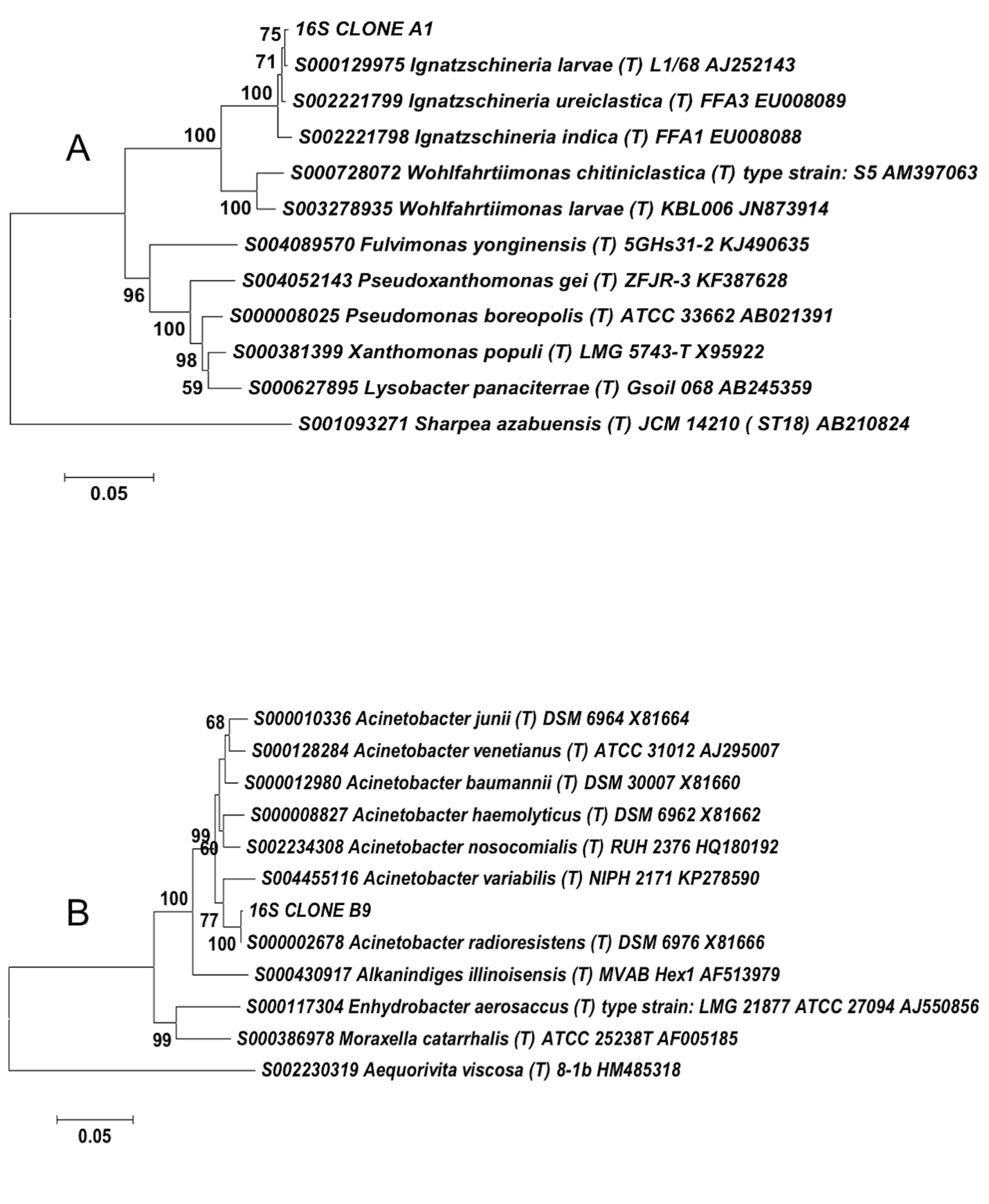
**

**Figure S4**

**
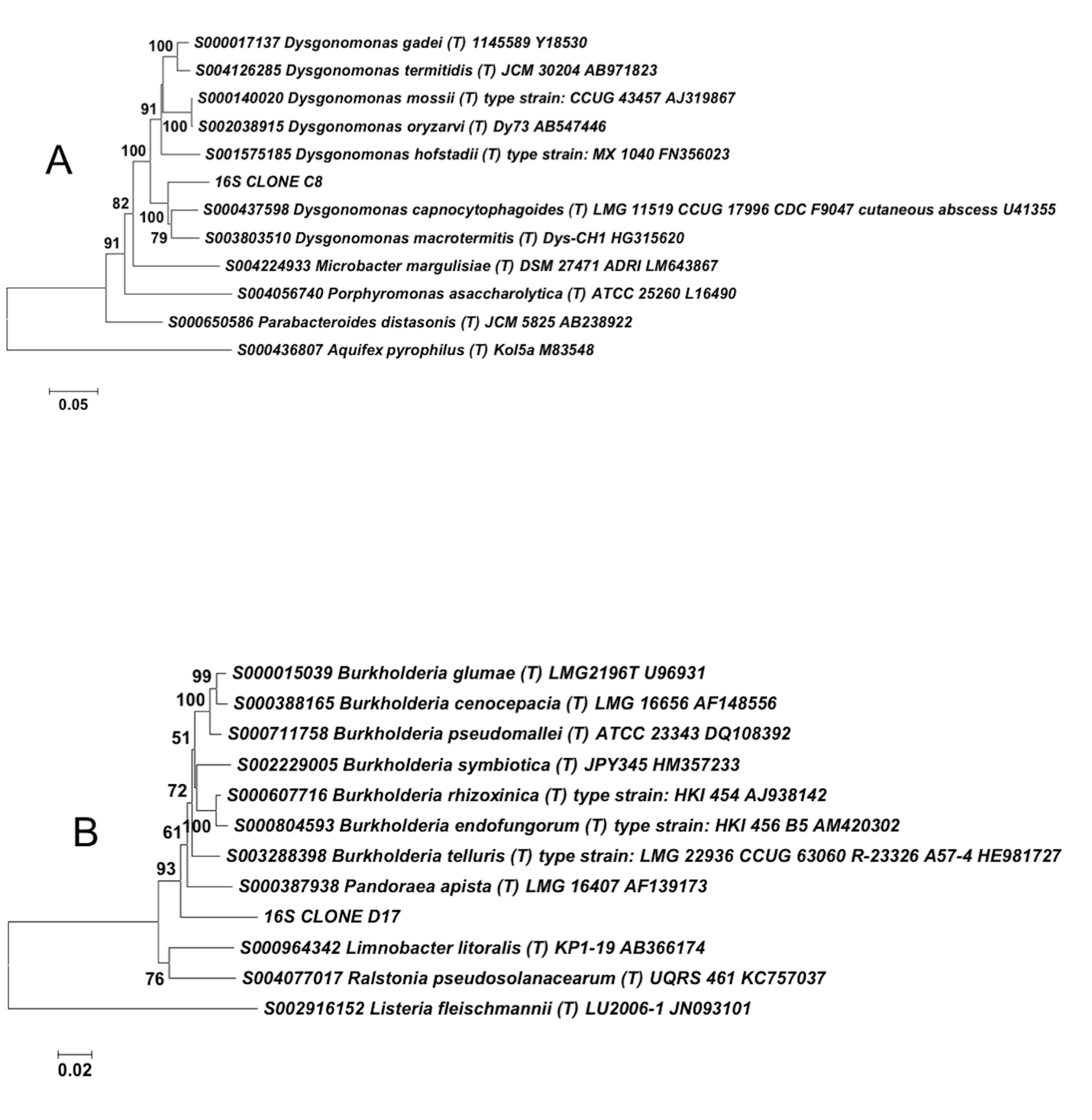
**

**Figure S5.**
